# Craniopharyngiomas in children: the pendulum moves again for an aggressive surgery—late complications and considerations with a recent series of 26 patients treated in Lyon

**DOI:** 10.1007/s00381-025-06815-3

**Published:** 2025-04-26

**Authors:** Pierre-Aurélien Beuriat, Alexandru Szathmari, Federico Di Rocco, Carine Villanueva, Lucie Bazus, Sara Cabet, Marina Veyrie, Carmine Mottolese

**Affiliations:** 1 Department of Pediatric Neurosurgery, Hôpital Femme Mère Enfant, 32 Avenue du Doyen Jean Lépine, 69677 Lyon Cedex, France; 2 UMR 5229, Institute of Cognitive Science Marc Jeannerod, CNRS, 69500 Bron, France; 3 Université Claude Bernard, Lyon 1, 69100 Villeurbanne, France; 4 Department of Pediatric Endocrinology, Hôpital Femme Mère Enfant, 69677 Lyon Cedex, France; 5 Department of Pediatric Radiology, Hôpital Femme Mère Enfant, 69677 Lyon Cedex, France

**Keywords:** Long-term outcome, Craniopharyngioma (CP) surgery, Transcranial approach, Pendulum

## Abstract

**Background:**

Craniopharyngioma (CP) surgery in children leads to high rate of recurrence, and morbid complications. Transcranial approach is the most frequently proposed surgical technique, but transsphenoidal endoscopic approach is also used. Pre- and post-operative complications of the tumor are well known, but early multidisciplinary management could improve the long-term outcome of these patients. The aim of this study was to analyse the risk factors for pre-and post-operative long-term complications in a series of patients operated with an aggressive removal for CP.

**Methods:**

A retrospective study of 26 children diagnosed with CP was carried out. The surgical total removal was possible in 88% of cases after the revision of the post-operative MRI realized in the first 48 h.

**Results:**

Children with hypothalamic involvement were more likely to have endocrine deficits and to be overweight or obese pre-operatively. They also had a higher risk of early post-operative complications, and late morbidities.

**Conclusion:**

Children with CP and strong hypothalamic involvement, have a higher risk of pre- and post-operative complications but complete removal is associated with a high rate of cure with global good neuropsychological results. Early multidisciplinary post-operative management should be reinforced to improve the long-term outcome but surgery with total removal can insure definitive acceptable clinical results.

## Introduction

Craniopharyngiomas is a rare benign tumor developing from neuro epithelial remnants of the Rathke pouch that is a remnant of the primitive pharynx [1–5]. Gaining surgical experience in craniopharyngioma surgery is difficult and surgical results are characterized by a high rate of sequelae preventing patients from having a good quality of life with worsening of the initial clinical picture [6–8].

The sequels of surgery are characterized by visual and endocrinological disturbances, motor and intellectual impairment.

Actually, the important improvement of diagnostic radiological tools with more efficient MRI—more efficient surgical tools, like the neuronavigation guidance system, the *Cusa Cavitron* and the endoscopic improved vision and overtime—and the significant improvement of the quality of paediatric anaesthesia have permitted the improvement of clinical results.

With current progress, several different strategies have been possible: a complete surgical removal, a partial removal followed by radiotherapy, intracystic treatment with local chemotherapy or gamma knife irradiation [1, 2, 6–12].

Our own strategies for management of craniopharyngiomas changed over the past 20 years and we moved from a surgical aggressive treatment in the years eighty, to an intracystic chemotherapy with bleomycin, and more recently, with alpha interferon for cystic craniopharyngiomas, reserving a surgical direct removal only for solid and calcified tumors or in cases of failure of the previous treatment.

Of great importance is the multidisciplinary management of children with the diagnosis of craniopharyngioma for the management of hormonal, hypothalamic and visual sequels and for the surgical follow-up.

We report the results of a last series of 26 paediatric patients with CP from Lyon, operated during the last 10 years with the aim to realize a total surgical removal. We analysed the factors influencing post-operative complications and long-term management strategies in patients treated with the pendulum of the surgical removal again oriented through an aggressive surgery.

## Material and methods

### Study design

Twenty-six paediatric patients with CP who underwent a first surgical resection at the Department of Paediatric Neurosurgery of the Women Mother Child Hospital of Lyon (France) between January 2010 and July 2023 were included in the present study and retrospectively analysed. Parents of children have been informed of the study by mail and could refuse to participate. The charts of the included patients were reviewed retrospectively. The study was approved by the Clinical Research Ethics Committee of the hospital (CSE-HCL – IRB 00013204; N°21_641).

The study was conducted at the paediatric neurosurgery and endocrinology department, neurosurgical ward and histological results were reviewed at the Pathological department and patients’ files were retrieved from the records departments in the Mother and Child Hospital in Lyon-Bron, France. The follow-up varied from 1 year to 11 years after treatment. Records of retrospective patients were retrieved.

### Inclusion/exclusion criteria

- Inclusion criteria
  - All patients with confirmed diagnosis and admitted with craniopharyngioma in the Woman Mother and Child Hospital in Lyon during the study period
- Exclusion criteria
  - Patients outside the above inclusion criteria
  - Patient not operated within the Mother and Child Hospital in Lyon

### Data collection

- Preoperative data including age at diagnosis, sex, height, weight, body mass index (BMI) and International Obesity Task Force (IOTF), clinical presentation, diabetes insipidus were considered. All patients had complete hormonal assays at diagnosis to evaluate if they have growth hormone (GH) deficiency (confirmed by IGF1 level <− 1DS and inhibition of growth velocity or insufficient GH secretion after pharmacological stimulation), central hypothyroidism (decrease free T4 level without an appropriate elevation of TSH concentration), central adrenal insufficiency (decrease level of cortisol at 8 a.m.). In the case of pubertal age, they have LH, FSH and sex steroid assays.
- MRI tumor characteristics (classification, maximal diameter, prior drainage of cyst, hydrocephalus) were collected. All pre-operative MRIs (with and without gadolinium) have been reviewed by an independent radiologist who determined the grade of the tumor according to the Puget classification [13].
- All patients had pre-operative ophthalmological evaluation with visual field, visual acuity and the fundus of the eye.
- Treatment: surgical and medical data were also extracted from the charts.
  - Preoperative treatment, prior drainage of cyst, surgical route macroscopic total or partial resection were collected from the study of the operative chart and the study of the first post-operative MRI realization in the first 48 h post-operatively. Patients with hydrocephalus first had VP shunt before the surgical direct approach.
  - Patients with preoperative cortisol deficiency and thyroid hormone deficiency were supplemented.
  - Two surgical approaches were realized: transcranial or transsphenoidal approach, according to the preoperative MRI observation.
- Follow-up: the patients were followed up at 1, 6 months and 1 year, 2 years, 5 and ten years. Endocrine hormone assays, auxological data and imaging examinations were performed at each follow-up visit.
  - Pituitary deficiencies, tumor recurrence were collected.
  - The term CP recurrence refers strictly to the evidence of tumor on neuroimaging studies after the evaluation of the complete surgical resection with the study of the post-operative MRI and of the operative chart and the negative postoperative follow-up with MRI study.

Neuropsychological assessments were carried out on the children at a distance from the surgery and collected in the article. Children under 16 years of age were tested with the WISC V scale of intelligence and children above 16 years of age with the WAIS IV. The same tests were used the verbal memory, the early memory system, the late memory and the late recognition, Attention test and quality of life questionnaire.

### Statistical methods

- Descriptive statistics were produced. Frequency of interested data has been expressed in percentage.

## Results

Twenty-six patients were treated from January 2010 to July 2023 for a craniopharyngioma. The age was comprised between 18 months and 18 years old with a median age at the moment of the diagnosis of 6.8 years. The follow-up varied from 1 year to 11 years with a median follow-up of 5.2 years.

Eleven patients were females (42%) and 15 were males (58%).

Symptoms at diagnosis: 12 patients (46%) presented neurological symptoms of intracranial hypertension, 5 (19%) ophthalmological troubles, 8 (30%) presented growth failure and 6 (23%) had a clear obesity.

In one patient, the diagnosis was an incidental discovery (deafness report). From endocrinological point of view, 17 children (65%) presented a growth hormone deficiency, 11 (42%) presented a thyrotropic deficiency, 10 (38%) presented an ACTH deficiency, 7 (14%) presented a gonadotropic deficiency, and 3 (11%°) had diabetes insipidus before surgery.

According to the classification of Puget, the MRI showed 6 patients in type 0, 9 in type I and 11 patients in type 2.

The pre-operative ophthalmological consultation showed an amputation of the visual field in 16 patients (53%) and an achievement of the visual acuity in 14 patients (53%).

Six patients (23%) were treated before the surgical approach with intracystic alpha interferon, that was effective at the beginning for all patients, but after all, escape to the treatment and surgery was decided. The median delay between the treatment and the surgical resection, related to progression of the tumor, was of 3 years varying from 1 year in three patients to 5 years in two patients. In two patients, the surgical removal was realized after 4 years.

Twenty-five patients (96.2%) were submitted to a surgical procedure by a transcranial approach while a transsphenoidal approach was realized in one patient (3.8%). Nine patients (34.6%) had a VP shunt for an active hydrocephalus before the approach to the tumor.

The surgical resection was evaluated by surgeons and a radiologist with a post- operative MRI realized in the first 48 h after the resection: it was complete in 22 patients (88%) and partial in 4 patients (22%).

### Genetic and molecular characterization

All our cases were reviewed by a paediatric pathologist and all were of adamantine type. The genetic study showed in only four cases the expression of B-catenin, and in two cases, the mutation of the CNNBI coding for the B-catenin that is normally expressed in the adamantine form.

### Treatment after surgery

Six patients (23%) were treated with proton therapy: two patients in whom the post-operative MRI showed a little residual nodule in the sellar region and four patients after a recurrence treated with a surgical resection, in two cases by a transsphenoidal endoscopic approach and by transcranial approach in the others two.

### Short-term complications

After surgery, 88% of patients had GH deficiency, 96% had thyrotropin deficiency and one patient had ACTH deficiency (14%). Diabetes insipidus was present in 96% of cases.

Seventeen patients presented in the post-operative course a triphasic hydroelectrolyte dysfunction characterized by an initial diabetes insipidus, a syndrome related to a realising of antidiuretic hormone followed by a persistent diabetes insipidus [14].

Eight (30.7%) patients presented isolated and persistent diabetes insipidus and 2 patients presented a salt wasting syndrome with a further decrease in sodium concentration about 10 days after surgery.

Three patients operated on for recurrence again presented with a triphasic syndrome similar to that following the first surgery.

### Long-term evolution

Six (23%) patients had recurrence during the follow-up and 4 had a rapid second surgery: the recurrences occurred 4 months, 6 months, 8 months, 1 year, 2 years and 4 months and 4 years and 6 months after first surgery.

Four patients had post operatory ophthalmologic complications.

From the endocrinological point of view, 1 year after surgery, 25 patients (96%) had GH, ACTH and TSH deficiency, 10 (38,4%) had hypogonadism and 25 (96%) had DI.

### Overweight and obesity

At diagnosis, 27% were already overweight, 8% obese i.e. 35% were overweight and obese. Six months after surgery, 17% were overweight and 33% obese (26 patients) i.e. 40% overweight and obese. After 1 year, 20% were overweight and 36% obese (24 patients) i.e. 56% overweight and obese; then after 2 years, 18% of the patients were overweight and 32% obese (23 patients) i.e. 50% overweight and obese after two years.

So the number of overweight or obese patients increases after surgery at 6 months (from 35 to 40% at 6 months, then 56% at 1 year). At 2 years, there was a discrete decrease from 56 to 50%;

At diagnosis, only five of the obese patients belonged to the type 2 of Puget and one to the type 1, while after the surgery, four patients of the type I, already in overweight became clearly obese and 8 patients of the 2. One patient with a type 0 presented an important tendance to the overweight because parents favourited the intake of food and the abstention to each type of sportive activity, thing that underline the importance of the management of these patients and their family.

### Neuropsychological evaluation

Twenty-five patients (96%) attended a normal or adapted school, while one patient was not yet of school age. Twenty-two patients (84%) followed a normal school program and three patients were able to follow a normal program with assisted scholar help.

Fifteen patients were examined and underwent neuropsychological evaluation between November 2022 and February 2024. They were tested with the Wechsler intelligence scale (WIPPSI IV (*N* = 1); WISC V (*N* = 13) or WAIS IV (*N* = 2)) according to age to evaluate processing speed, fluid reasoning, verbal comprehension, working memory and global intelligence. All patients had index scores in the normal range.

Patients were also given an attention test (ZOO map, BADS and Tea-ch (score! and sky search)), which they all completed without difficulty. Two patients had difficulty for BADS test.

For the memory assessment (children memory scale or RLRI 16 test (French adaptation of the Free and Cued Selective Reminding Test)), 3 out of 7 patients had a deficit score (42%).

These patients were also given questionnaires. Concerning anxiety (Hospital Anxiety and Depression scale), only one patient in five had a pathological score (20%). For quality of life (PedsQL Wellbeing), we received responses from 13 patients with good results in 75% of cases and 15% pathological for the overall score. Three children out of 13 (23%) had pathological scores for psychosocial and psychophysical quality scores (2 only physical health, 2 only psycho social deficits and only one patient had both). For the fatigue scale (Peds QL Fatigue), 3 out of 12 patients had a pathological fatigability score (25%).

With the behaviour rating inventory of executive function (BRIEF), we found for 11 patients: 4 pathological scores for the emotional control subtest (36%), 3 deficits to start an action “initiation activity” (27%), 5 pathological scores for task control (45%). And 5 out of 12 deficits scores for metacognition condition (knowledge of one’s own competence and abilities) (41%), and 4 out 12 patients (33%), for flexibility (ability to switch from one task to another). Emotional control was also difficult for 4 out of 12 patients (33%).

## Discussion

Treatment of CP still remains a challenge for neurosurgeons and especially for paediatric neurosurgeons.

Results of large series published over the last 20 years showed visual troubles in 70% of patients, endocrine problems in 95%, neuropsychological troubles in 50% patients, and the incidence of recurrences in 25% of cases [15]. Thomson underscored better results after the years 1996 with the advent of major technological advances [16].

Of the 55% of patients with pre-operative visual disturbances, only 12% of cases had normal visual acuity after surgery [17].

While to-day surgery remains a very important step for treatment, the deep location and the important anatomical structures such as the optic pathways, the hypothalamus, the carotid and basilar arterial system and the pituitary gland and the pituitary stalk are responsible for clinical sequelae [15–17].

Many surgical approaches have been proposed in the recent years but the main problem remains the limits of the surgical resection varying between a complete to a partial removal followed by radiotherapy to decrease sequelae.

The reasons for a complete surgical removal have been advocated by many authors [16, 18–24]: a benign tumor completely removed provides the best chance of durable cure and of disease control with an acceptable quality of life, associated to a better survival rate and progression-free survival [4].

Hydrocephalus or a VP shunt have a significant impact not only on overall survival but also on progression-free survival [4].

Lesions of the antero-medial and lateral hypothalamus, controlling satiety and food intake, can be responsible of hyperphagia and loss of the circadian rhythm of food intake increasing obesity or in some cases, loss of appetite resulting in refusal to eat, which can lead to the death [8, 25, 26].

The role of hypothalamus in determining cognitive and behavioural impairment has also been emphasized [8, 25, 26] and to avoid hypothalamic lesions a strategy renouncing to a complete resection, at all costs, has been abandoned for a more conservative attitude adopting a partial removal followed by radiotherapy.

Many authors reported an high rate of disease control with partial resection and radiotherapy with results at least as good as those of complete removal in terms of survival but associated to a better quality of life [3, 27–35].

On the contrary, Zuccaro and Di Rocco underscored that with total removal patients were able to attend school whereas only 62% of patients with a sub-total resection and radiotherapy were able to attend school [15, 17].

No significant differences were observed in 5 and 10 years’ overall survival and progression-free survival between GTR and STR followed by adjuvant therapy [36–38].

The adjuvant radiotherapy is important because a sub-total removal without adjuvant radiotherapy ensures poorer overall survival and progression-free survival with a rate of recurrences in 63% of cases [36–38].

In our last series patients were treated with an aggressive surgical resection in 88% of patients. The surgical approach was transcranial in 96% of cases related to the extension of the tumor and its volume; a transnasal transsphenoidal endoscopic approach was adopted in only one case with intra sellar and suprasellar extension strictly on the mid line.

Complete removal, in experienced hands, permits the cure of this benign tumor with good results avoiding radiotherapy. Our attitude of an aggressive surgical removal of CP was encouraged because, alternative strategies, as local intracystic treatments, with bleomycin and interferon alpha, were not more available because bleomycin was toxic for the CNS and the interferon alpha was no more available for intracystic use. Consequently, the pendulum moved again to an aggressive surgery with a good quality of life in 96% of cases.

### Surgery

The surgical resection of CP is a challenge for experienced paediatric neurosurgeons. The rarity of the lesion (two new cases per millions per years in the USA and in France) [56, 57] and, on the other hand, the deep localization with close relationships with nervous and vascular structures make at risk the quality of life of patients.

Many surgical approaches have been proposed: subfrontal, pterional, frontotemporal, sub-temporal, interhemispheric and transcallosal each of them with specific advantages and disadvantages.

We prefer the subfrontal pterional approach that permits the exposition of the four basal triangles whose exposition is imperative to remove craniopharyngiomas mainly when they are of great volume as frequently in children.

**Fig. 1:**
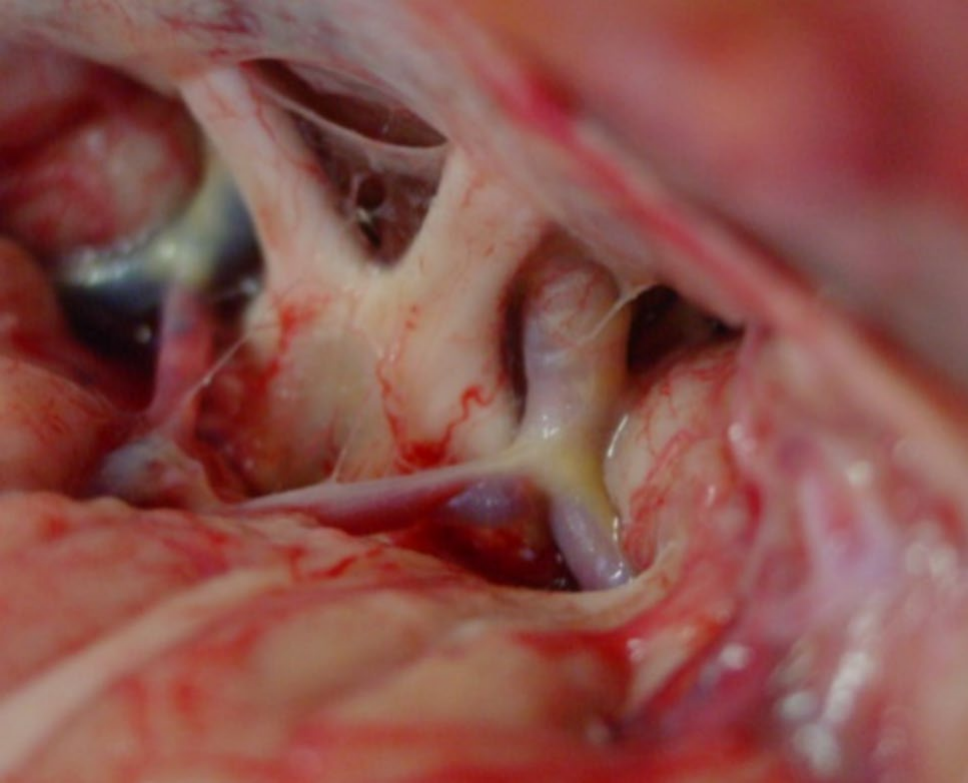
Anatomical dissection showing the surgical exposure with the subfrontal pterional approach

The four triangles are the opto-carotid, the retrocarotid, the interoptic triangle that, when the optic nerves are long and chiasma retro fixed, give a very large space for the dissection; and the fourth triangle delimited by the carotid bifurcation that is very interesting for removal of the upper portion of very huge tumors (Fig. 1).

The transfrontal transventricular approach through the third ventricle, for craniopharyngioma resection, has for us limited indications because craniopharyngiomas of the third ventricle are very rare, only 4% of cases in the series of Yasargyl [58], and because craniopharyngioma growing upwards raised the floor of the third ventricle exposing the floor to damages for the removal of the tumor: we used it in our experience only in eight cases [24] and in no case of this last series.

The opening of the lamina terminalis represents a good door for resection of craniopharyngiomas when the chiasm is ante fixed for the presence of short optic nerves.

**Fig. 2:**
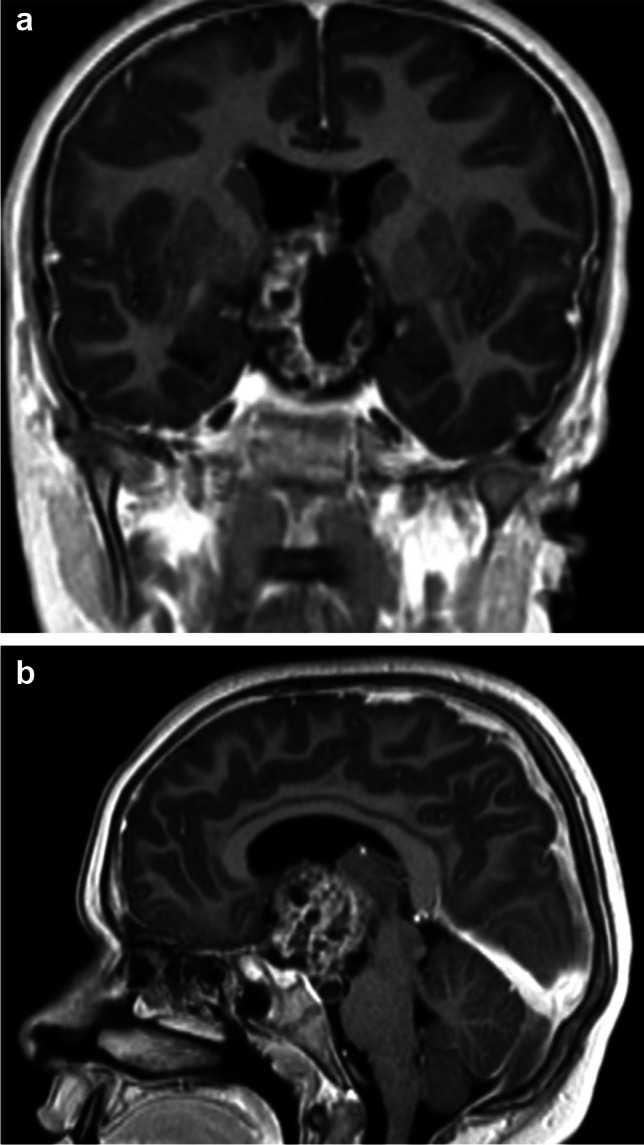
**a** and **b** Pre-operative MRI of a mixed form of craniopharyngioma before the surgical treatment

Crucial is the dissection of CP at level of the optic pathways and the experience of the surgeon is important to preserve the function of the optic pathway. In our last series of 26 patients, 16 patients (63%) before the surgical procedure had visual problems, while after surgery, only 9 patients presented visual troubles (35%) translating the fact that in 7 patients (27%), the decompression favourited the visual improvement, while in the others patients, the decompression, could not be effective to recover deficits appeared from long time (Fig. 2a and b).

**Fig. 3:**
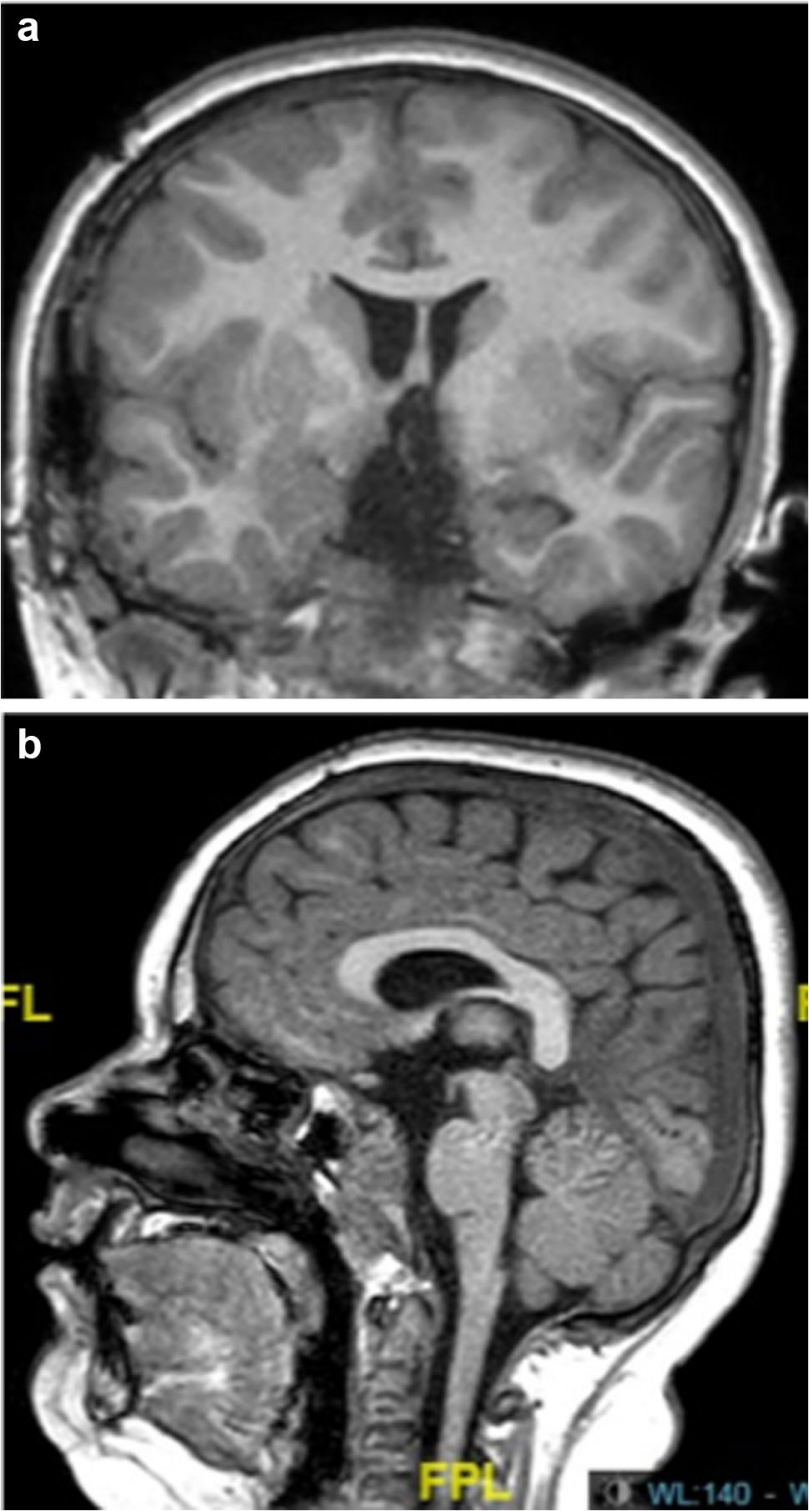
**a** and **b** Post-operative MRI showing the complete removal of the tumor

Lesions of hypothalamus with repercussions on the hypothalamo-pituitary axis and on behavioural troubles with difficulty to control the thirsty, the hunger, the aggressiveness and memory and school difficulties represent also a critical problem (Fig. 3a and b).

The lesions of the hypothalamus are not only related to the surgical trauma of the dissection but mainly to the ischemic lesions in relation with the occlusion of the perforating vessels originating from the posterior communicating arteries [24, 58, 59] frequently object of inopportune coagulation.

The CP is an extra-arachnoid tumor and respecting the arachnoid plane of dissection it is possible to avoid problems with the dissection of posterior portion with the basilar artery trunk. The perforating vessels of the basilar trunk are headed back, in a plane posterior to that of the tumor with a posterior direction through the mesencephalon and consequently can be spared.

The endoscopic approach has permitted to reduce the exposition of nervous structures for craniopharyngiomas removal favouring a great enthusiasm for it.

Surgery of CP with endoscopic endonasal transsphenoidal approach in children, in our opinion, should to be addressed only for lesions developing strictly on the mid line and that remain exclusively under the diaphragm sellae without lateral extension and without the need of extended opening of the skull base, and consequently, it has been realized in only 4% of our cases.

For huge tumors and for tumor with a lateral development, as frequently observed in paediatric cases, the endoscopic technique is not indicated for the difficulties to close the skull base and for the high risk of infectious complications with a rate of mortality for fulminans meningitis with 8% of mortality [60, 61].

Mazzatenta reported a paediatric series treated with endoscopy with a rate of peri-operative mortality in 4% of cases and a rate of recurrences of 19% [61].

In our reported series, we treated only a patient with an endoscopic resection for a primary treatment and two patients for an intrasellar recurrence but, for us, endoscopy remains an important tool, after the microsurgical dissection, to explore the operative field to discover pieces of tumor left in place that, removed, increase the rate of complete resection, reducing the risk of recurrences. We recommend the endoscopic exploration in each case at the end of the microsurgical resection.

### Radiotherapy in treatment of craniopharyngiomas

Radiotherapy after partial resection of CP has been recommended and many authors reported favourable results in 2/3 of patients [39]. Radiotherapy is not deprived of complications: paediatric patients present social and emotional functioning performances lower than a control group, difficulties for learning, difficulties for behavioural control and problems for controlling emotions and concerns for physical appearance as reported by Heinkes [40].

Radiotherapy becomes, in the last years, the standard of care in case of subtotal removal [38, 41] and new methods of irradiation have permitted to improve and to reduce the doses of irradiation to the adjacent structures: hypothalamus, optic pathways, the pituitary gland, the carotid arteries and the medial temporal structures. Conformational radiotherapy, proton therapy, intensity modulated radiotherapy and fractionated stereotactic radiotherapy have been used to reduce the risk of sequels [42].

The use of stereotactic radiosurgery for lesions of small volume has also been proposed with cautious because high dose applied in a single dose can be responsible of lesion of vital structures [42, 43].

Only 6 patients of our series for a recurrence have been treated with a surgical resection followed by proton therapy to decrease the doses on nervous and vascular structures but, it seems us, better to avoid radiotherapy when a complete surgical resection is possible as in 76% of our cases [32].

### Molecular pathology

In the last years, also, craniopharyngiomas have made the object of molecular studies with the aim to find targets that could permit a tailored medical treatment that could be an alternative to surgery in the next future.

Two different subtypes of craniopharyngiomas have been described the adamantinous (ACPs) predominant in paediatric cases and the papillary craniopharyngiomas (PCPs). The average age of incidence is regulated by two pic of age, one between 5 and 12 years old in children and another, in adult patients, between 50 and 70 years old [7].

Papillary CP occur rarely in paediatric age and their average of diagnosis is between 44 +/− 15 years and have a better 5 years’ survival rate and a less aggressive disease behaviour [44].

The genes involved in the pathogenesis of craniopharyngiomas can conditioned their medical treatments.

In ACPs the Wingless pathway (WNT pathway) activation seems to be regulated by the gene CTNNB1, encoding B-catenin that are activated in more than 2/3 of tumors in recent studies [45]. In other patients, also, the MAPK/ERK pathway has been demonstrated opening novel therapeutic strategies with the suppression of this pathway with chemical agents like MEK inhibitor trametinib [46].

In PCPs, the subgroup BRAF V600E mutation has been found in 90% of cases [45, 47]. The BRAF system is a proto-oncogene encoding serine-threonine kinase involved in growth factor signalling and regulation. Its mutation results in an active form that promotes the cell proliferation and tumor growth.

These mutations in PCPs pushed to treat some patients with targeted therapy in cases of recurrences or for reducing the volume of the tumor [47, 48].

The expression of the Beta catenin was present in four of our patients and in one the mutation of the gene CNNBI that code for the Beta catenin.

The genetic studies can be useful for tailored treatment of cystic types of papillary craniopharyngiomas in adults to reduce their volume or in case of huge recurrences but, until now, there are no treatments effective for cure paediatric craniopharyngiomas.

### Intracystic chemotherapy

To reduce risks and sequels of surgery we practised intracystic chemotherapy for craniopharyngiomas after the implantation of an Ommaya reservoir [49].

At the beginning of our experience for cystic recurrences and, successively, after the good results obtained, for primary cystic form of craniopharyngiomas.

Bleomycin, introduced by Umezawa for treatment of cerebral cystic lesions [50], was reported by Takahashi in 1985 with good results for treatment of craniopharyngiomas [51] and successively by Broggi and Lapras and Mottolese [49, 52].

The action of bleomycin was at level of the lattico-deidrogenasis system blocking the duplication of the DNA and consequently inducing the death of cells [24, 49].

The toxicity of the drug for the SNC and a severe complication with the injection of a toxic doses responsible of blindness in a patient pushed us, after the experience of Di Rocco and Cavalheiro, to use the interferon alpha [15, 53].

Interferon alpha is not toxic for the central nervous system (CNS) but its effects were limited in the time also if permitted to delay surgery, the worsening of pituitary insufficiency with surgery and the onset of post-surgical complications.

In 7 patients treated with interferon alpha, the median delay between the treatment and the surgical resection for the progression of the tumor was of 3 years varying from 1 year in three patients to 5 years in two patients. In two patients, the surgical removal was realized after 4 years. The mechanism of action of the interferon alpha seems to be mediated by an inflammatory reaction [15, 54].

The variable efficacy and the limited effect in time of the drug could be related to different genetic tumoral constitution responsible of a different response to a programmed inflammatory reaction induced by the drug.

Actually, the interferon alpha is not available for intracystic administration, and consequently, this fact pushed us again to a surgical removal of craniopharyngiomas. The subcutaneous injection of the peginterferon alpha- 2b [55] has also been proposed but we have no experience with this drug. We think that in the future, the possibility to dispose of effective drugs could completely change the strategy for treatment of craniopharyngiomas.

### Recurrences

The recurrences rate after total surgical removal of craniopharyngiomas have an incidence in literature varying from 4 to 30% [21, 24, 57, 62–65, 67, 68].

Osborn showed a proportional relationship between recurrences and the volume of the tumor: for tumor with a diameter inferior to 5 cm. The rate of recurrences was of 20% while for tumor with a diameter > to 5 cm. The rate of recurrence increased to 80% [69].

In our last series, the recurrence rate after a total removal was of 20%. Four patients with a residual volume after the first surgery were treated with a new surgical resection in two cases by a transsphenoidal and with a transcranial approach for the other two, with a complete removal followed by proton therapy.

All these cases did not show a new progression after a median follow-up of 3 years.

In our last series, 20 patients have been treated with only surgery: consequently, for us, the complete removal of craniopharyngiomas can be the aim of surgery because a complete resection permits a definitive cure of a benign lesion also with endocrinological, visual and behavioural troubles that are already present, at diagnosis, in 53% of our cases. The good results as the good neuropsychological evolution of our patients with surgical complete resection confirms that also large craniopharyngiomas can be treated with a complete resection in contrast with the actual policy to realize partial resection to reduce the incidence of hypothalamic severe lesions reported in literature.

Lesions of hypothalamus can be prevented with the respect of the perforating vessels of the posterior communicating arteries [24].

The respect of the arachnoidal sheaths guarantee the protection of vascular and nervous structures favouring a good quality of life after surgery.

A meticulous atraumatic dissection at level of the hypothalamus and of the optic pathways reduce the risks of post-operative clinical aggravation as showed by the improved results concerning the visual acuity and the neuropsychological evolution of our patients associated also with good scholar results in 96% of cases.

The high number of patients with a normal life, attending a normal school program, are witnesses of the value of surgery and the progress of the surgical techniques in the last years.

Out of 26 patients considered, 15 were analysed in the last year with exhaustive neuropsychological tests; we observed a contrast between the results of the performances obtained, and the difficulties encountered in daily life.

The majority of these children are in great social distress, leading to withdrawal and even, in more severe cases, dropping out of school.

While the main hypothesis was to explain these difficulties by cognitive deficits resulting from the brain damage, the data obtained suggest more socio-psychological difficulties without cognitive impairments also in the patients with type II craniopharyngioma according the classification of Puget.

The classification of Puget, in our sense, translates only the fact that larger tumors are more difficult to remove but does not explain why patients with large post-surgical void at level of hypothalamus present a good clinical evolution and others with 0 and type I can have a bad evolution. At level of hypothalamus the surgical dissection has to be cautious but, in our experience, it should be realized whenever possible avoiding coagulations.

The fact that obesity, in our series, improves with the time means also that obesity represents a complex problem that is related not only to lesion of the hypothalamus but also to a complex therapeutical program concerning also social and family education necessary to improve results.

Our study demonstrates the shortcomings in the psychological follow-up of these children and their families. Improving the follow-up of these children through group therapy, in-depth psychological monitoring and monitoring of diet and weight management would be relevant to their care. With better care, these children could more fulfilled in their school careers. The overweight and obesity can be better taken in care using better protocols of monitoring avoiding unnecessary food intake with better social and educative programs not only for patients but also for their family. For this reason for us, it can be accepted that the pendulum of the surgical removal can move again, in experienced hands, towards an aggressive surgical resection until advances in biomolecular studies offer the possibility to switch to medical treatments with drugs administration, at the condition, to avoid serious toxicity and complications related to radiotherapy mainly in very young children. For us, the concept is valuable of safe maximal surgery, whenever it is possible, for a complete resection for craniopharyngiomas.

### Endocrinological considerations

The consequence of surgery for craniopharyngiomas is dysregulation of the hypothalamo-pituitary axis which affects 97% of patients. An early post-operative complication is triphasic syndrome.

The incidence of triphasic syndrome was more frequent in our experience than in others of literature [70, 71]. A great deal of clinical experience is needed to avoid this phenomenon, which in the post-operative period requires to avoid very severe fluid restriction at the outset, to compensate for water loss, and which needs the cautious use of antidiuretic hormone to avoid the conflict with SIADH syndrome, which can be very dangerous for the clinical evolution of patients. We emphasize the role of paediatric endocrinologists in collaboration with the intensive care physicians in limiting the incidence and effects of this syndrome.

It is true that the relationship between the tumor and the floor of the third ventricle and the large volume of the tumor are significant factors in the appearance of this syndrome.

**Fig. 4:**
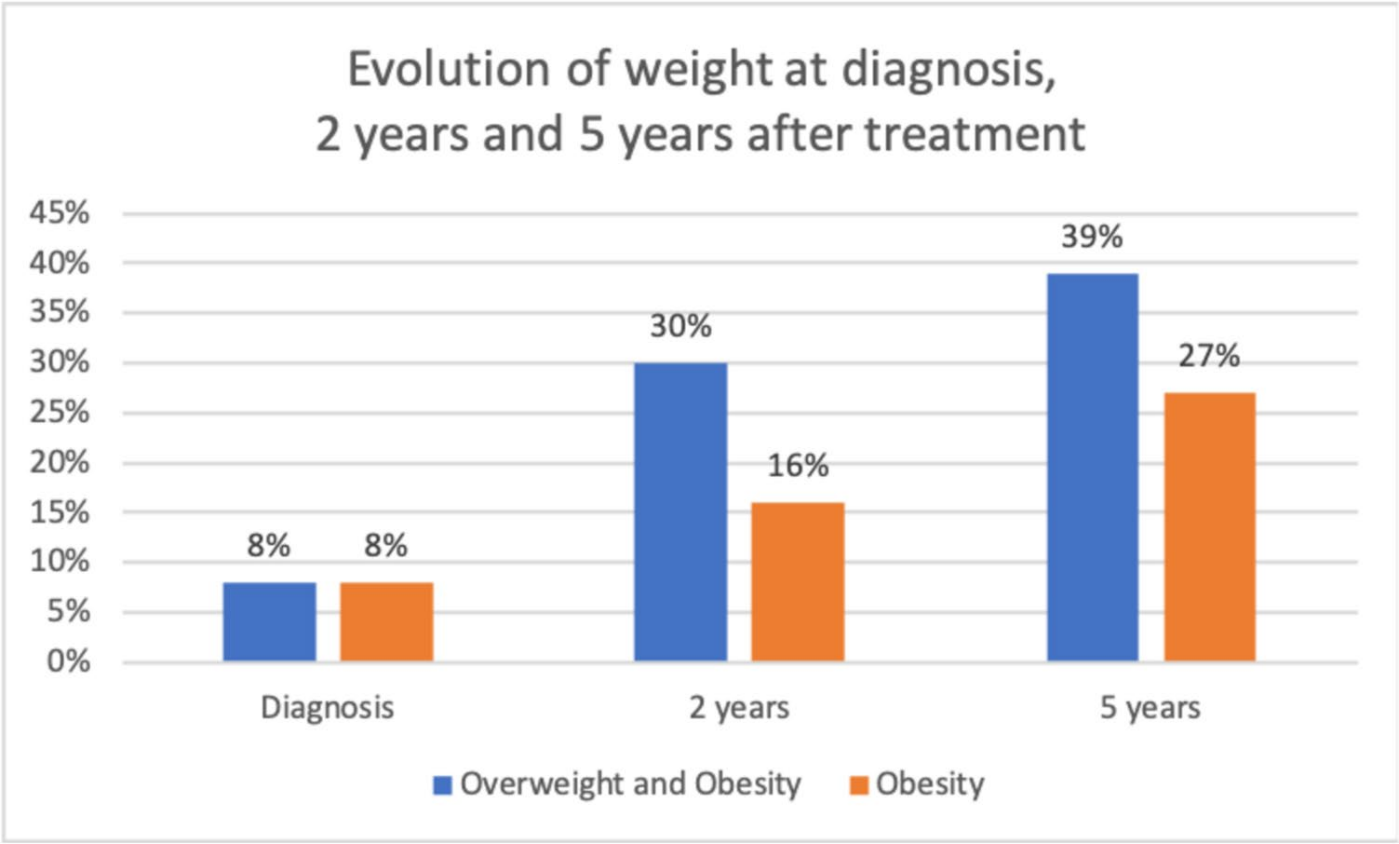
Bar graph showing The evolution of the incidence of the overweight and obesity after diagnosis

The number of overweight or obese patients increases after surgery at 6 months (from 35% at diagnosis to to 40% at 6 months), then at 1 year (56%). At 2 years, a decrease from 56 to 50% was observed; The role of the multidisciplinary management (dietetic follow-up, adapted physical activity instructor, psychologist, paediatrician, paediatric neurosurgeon) introduced in our department 8 years ago seemed very important to control the overweight and obesity (Fig. 4).

## Conclusions

Craniopharyngioma remains a difficult tumor to treat in the paediatric population.

The role of surgery is still a topical issue in paediatric craniopharyngiomas. It is always difficult to decide intraoperatively whether to completely remove the tumor or to stop resection and proceed with partial removal followed by radiotherapy.

Surgery requires special expertise to respect nervous and vascular structures, which represent the main risk factors for sequelae and sometimes the cause of catastrophic results. Our latest cohort shows that complete resection is possible, even if associated with endocrinological (96%) and visual sequelae (23%) but with good neuropsychological evolution with a safe maximal surgery.

Surgery in experienced hands can be the treatment of craniopharyngiomas also if the extent of resection should always be related to a pendulum that shall move in relation with the experience and the expertise of surgeons, the extension and the volume of the tumor, the possibility in the future to find effective pharmacological treatments to preserve the aggravation of function already affected when tumors are discovered.

## Data Availability

No datasets were generated or analysed during the current study.
